# Tuberculosis in North Carolina: trends across two decades, 1980-1999.

**DOI:** 10.3201/eid0707.010739

**Published:** 2001

**Authors:** H M Salihu, E Naik, W F O'Brien, G Dagne, R Ratard, T Mason

**Affiliations:** University of South Florida, Tampa, Florida 33612-3805, USA. hsalihu@hsc.usf.edu

## Abstract

In North Carolina, we analyzed cumulative data for tuberculosis (TB) from 1980 through 1999 to determine trends in incidence, population subgroups at risk, and implications for health policy- makers. The overall incidence rates declined significantly over the study period (p = 0.0001). This decline correlates strongly with an increase in TB patients receiving directly observed therapy. Males have approximately twice the risk for disease, and persons >65 years of age are at the highest risk. For every Caucasian with TB, six blacks, six Hispanics, and eight Asians have the disease. TB incidence rates are declining in all other population subgroups but increasing in foreign-born and Hispanic persons.


### Tuberculosis in North Carolina: Trends Across Two Decades, 1980–1999

**Hamisu M. Salihu,* Eknath Naik,* William F. O'Brien,* Getachew Dagne,* Raoul Ratard,† and Thomas Mason***
*University of South Florida, Tampa, Florida, USA; and †North Carolina Department of Health and Human Services, Raleigh, North Carolina, USA

In North Carolina, we analyzed cumulative data for tuberculosis (TB) from 1980 through 1999 to determine trends in incidence, population subgroups at risk, and implications for health policy-makers. The overall incidence rates declined significantly over the study period ($p = 0.0001$). This decline correlates strongly with an increase in TB patients receiving directly observed therapy. Males have approximately twice the risk for disease, and persons ≥65 years of age are at the highest risk. For every Caucasian with TB, six blacks, six Hispanics, and eight Asians have the disease. TB incidence rates are declining in all other population subgroups but increasing in foreign-born and Hispanic persons.

According to estimates of the World Health Organization (WHO), one third of the world's population is infected with tuberculosis (TB) (1). During the 1990s, approximately 90 million new cases have developed worldwide (1). In the United States, from 1953 through 1984, the incidence of TB declined an average of 5% per year, but increased by 20% during 1985 through 1992 (2). The nationwide peak in incidence of TB in 1992 led to renewed public commitment and investment of resources. Simultaneously, scientific investigations, especially at the national level, have resulted in interesting and useful findings for policy-making. However, such strategies, based on evidence from pooled data from all U.S. states and territories, may not necessarily be effective in certain regions of the United States, where characteristics of TB patients may differ substantially from the national pattern. Additionally, state and local health departments involved directly with TB prevention programs may require evidence-based information derived from locally available TB records, which more accurately represent the realities of TB disease in that locale. In this study, we describe the trends in TB incidence in North Carolina and the sociodemographic characteristics associated with elevated risk for the disease.

### Materials and Methods

Suspected or confirmed cases of TB are reportable by law in North Carolina. Cases reported to the state health department are verified according to Centers for Disease Control and Prevention (CDC) criteria (3). Confirmed cases are then registered, investigated, and managed according to a standard protocol. The complete information, including follow-up status, is recorded in the CDC Report of Verified Case of Tuberculosis (RVCT) form and forwarded to CDC electronically. The state health department also maintains all TB case records in its own database, which is the source of our data for this study. All confirmed incident cases of TB reported in North Carolina from 1980 to 1999 were considered in our analysis.

TB incidence rates (cases per 100,000) for North Carolina were computed from 1980 through 1999, allowing comparison of TB trends between the 2 decades (1980 to 1989 vs. 1990 to 1999). For comparison with national TB incidence rates over time as well as the ranking of North Carolina among other U.S. states, territories, and the District of Columbia, we included national TB cases and incidence rates from 1980 to 1999. In 1993, the state TB surveillance system was restructured and funding was increased after a nationwide peak in TB incident cases was observed. In determining whether the number of TB cases in the state has decreased substantially as a result of this investment of resources, a comparison of incident TB cases from 1980 to 1989 versus 1990 to 1999 can only underestimate any improvement. Consequently, any observed decline must be considerably important to be captured by this conservative approach. For the rest of the analysis, only information on TB cases from 1982 to 1999 was used because data were incomplete for most of the demographic variables before that time. The variables coding for country of origin and directly observed therapy (DOT) were added to the CDC RVCT form in 1993, so analysis involving these factors covers only 1993 to 1999.

The yearly rate of TB cases in the state was calculated by dividing the total incident TB cases by the 1990 population census or estimate for that year and multiplying by $10^5$. The denominators were obtained from the state census bureau. The national TB rates for the whole United States for the period of study were extracted from yearly reports in MMWR, which provide TB cases and rates by state (4-8). We then computed the average national rates after subtracting the contribution from North Carolina for each year before comparing the two.

In estimating the rate of TB for a given sociodemographic factor, data were adjusted for age by the direct method of standardization. For each variable, the incidence density rate was computed by dividing TB cases by the expected

Address for correspondence: Hamisu Mohammed Salihu, Department of Biostatistics and Epidemiology, College of Public Health, University of South Florida, 13201 Bruce B. Downs Blvd, Tampa, FL 33612-3805, USA; fax: 813-974-4719; e-mail: hsalihu@hsc.usf.edu person-years and multiplying by $10^5$. Using the PROC GENMOD in SAS, we generated incidence-density ratios and their 95% confidence intervals by maximum likelihood estimation, assuming a Poisson distribution for our data, namely, that the probability (Pr) of TB cases (y) per $10^5$ person-years is equal to some number r is given by

$$Pr(y=r)= \lambda e^{-\lambda}/r!$$

Where λ is the expected value (mean) of y and r!=r(r-1)(r-2)...(2) (1).

### Statistical Analysis

Data were analyzed with SAS software (version 6.12). Incidence rates of TB (expressed as TB cases per 100,000) in the United States over the study period were compared with those of North Carolina by two-sample t-test. Paired sample t-testing was used to assess differences in TB incidence rates in North Carolina between the two decades (1980 to 1989 vs. 1990 to 1999), as well as the national ranking of the state during the two periods. The underlying assumption is that, since data for the two periods were obtained from the same population source, they are correlated. An overall regression equation linking year and TB incidence rates in the state was modeled to determine the amount and direction of change in rates across the 2 decades, and the best-fitting slope was plotted. In determining the best predicted trajectory, a quadratic in addition to the linear term (of the independent variable of year), was added to the model, yielding a curvilinear figure. A similar procedure was performed for the selected sociodemographic factors using the linear model only. A trend in the proportion of TB patients receiving directly observed therapy (DOT) was compared with the corresponding yearly incidence rates of TB, as well as rates of therapy completion, estimated by Pearson's correlation coefficient. All tests of hypothesis were two-tailed, with a type 1 error rate fixed at 5%.

### Results

From January 1980 to December 1999, 13,564 incident cases of TB were recorded in North Carolina, for a yearly average of 678 new cases. From a peak of 1,066 cases in 1980, TB cases have steadily declined, with new TB cases for 1999 reported at 488, a decline of 54.2%. Comparison of trends over time in North Carolina TB rates with the U.S. national rates shows that both have declined continuously since 1992, the year the national TB incidence rates peaked after a continuous decline in the 1980s. The rate of decrease in the two populations was approximately the same (t = 0.98, p = 0.34). Analysis of the data for the national ranking of North Carolina across the 2 decades shows a significant improvement (p = 0.003) in the state's ranking in the second decade (1990 to 1999) compared with the first (1980 to 1989). From being third worst nationwide in 1980, the state now is in 17th position. A similar significant improvement was observed when rates were the comparative indices between the 2 decades (p = 0.0001). The regression coefficient for the trend in incidence rates over time shows that for each additional year since 1980, the rate of TB has declined by approximately 0.5 case per 100,000 population (p = 0.0001) (Figure 1).

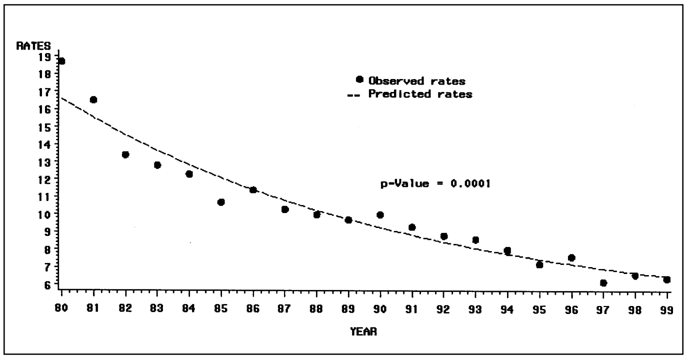

Figure 1. Incidence rates of tuberculosis, North Carolina, 1980-1999. (Rates are per 100,000)

### Sociodemographic Distribution of TB Disease (Table 1)

In general, males are twice as likely to have TB than females (Table 1); this 2:1 sex ratio has been constant over both study periods. A test of equality of variance over the study period for the two groups strongly supported equal deviations independent of general trends in incidence rates (F = 1.01, p = 0.99). However, the distribution of male:female ratio differs by race, ethnicity, and age group. Among Asians, males and females have the same risk for TB disease (1:1), whereas among Hispanics and Native Americans the proportion of males with the disease is three times that of females. Blacks, whites, and non-Hispanics have a 2:1 sex ratio. In both U.S.-born and foreign-born persons, males have twice the risk of females. At birth and up to the age of 24, there is no difference in risk by sex. Thereafter, more men contract TB than women, reaching a 2:1 ratio in the age group 25 to 44.

Table 1. Tuberculosis incidence density rates and ratios in North Carolina, by sociodemographic characteristics (1982 to 1999)

| Variable | Incidence Rate[a] (per $10^5$ person-years) | Rate Ratio | 95% Confidence Interval |
|---|---|---|---|
| Sex | | | |
| Female | 5.88 | 1.00 | 2.07-2.24 |
| Male | 12.70 | 2.16 | |
| Age Group[b] | | | |
| <15 | 1.51 | 1.00 | |
| 15-24 | 2.54 | 1.76 | 1.53-2.0 |
| 25-44 | 7.30 | 5.00 | 4.53-5.60 |
| 45-64 | 13.45 | 9.20 | 8.24-10.22 |
| ≥65 | 26.37 | 18.30 | 16.41-20.32 |
| Race | | | |
| White | 4.14 | 1.0 | |
| Black | 25.53 | 6.20 | 5.93-6.42 |
| Asian | 35.95 | 8.52 | 7.75-9.38 |
| Native American | 4.90 | 1.20 | 1.07-1.48 |
| Ethnicity | | | |
| Hispanic | 26.80 | 2.90 | 2.66-3.25 |
| Other | 9.10 | 1.0 | |
| Country of birth[c] | | | |
| Foreign-born | 10.70 | 1.0 | |
| US-born, all | 6.9 | 0.64 | 0.58-0.70 |
| US-born, blacks | 19.80 | 1.83 | 1.66-2.03 |

[a]Age-adjusted.
[b]Chi-square for trend = 7.66 (p = 0.006).
[c]1993-1999 only.

This trend reaches a peak in the age group 45 to 64, when for every woman contracting the disease, three men are affected. After the age of 65, the sex ratio returns to the 1:1 proportion observed at birth and childhood (<15 years).

The age distribution of patients with TB disease in North Carolina is negatively skewed, with a median age of onset of 51 years and a preponderance of cases in the older age groups. The highest risk for TB in the state is among the elderly (≥65 years of age). This group has 18 times the risk of children <15 years of age, the age group with the lowest risk (Table 1). With respect to trend over time, the incidence rates of TB have decreased in all age groups (Table 2). The most impressive decrease is in the elderly population (≥65) to approximately 3 cases per $10^5$ person-years. The rate of decline in those <25 years of age is insignificant.

Blacks, Hispanics, and Asians have the highest TB rates in North Carolina (Table 1). For every white person affected by TB disease, six blacks, six Hispanics, and eight Asians have the disease. Among blacks, TB incidence rates have steadily decreased since 1982, from 37.3 cases per $10^5$ person-years in 1982 to 16.2 cases per $10^5$ person-years in 1999. This decline is highly significant, corresponding to 1 case per $10^5$ person-years ($p < 0.0001$) (Table 2). The incidence rates among Asians have fluctuated since 1982. The highest rate, 59.6 cases per $10^5$ person-years, was recorded in 1982, and the nadir, 27.0 cases per $10^5$ person-years, occurred in 1990. The latest TB incidence rate (1999) for Asians is 47.4 TB cases per $10^5$ person-years. Despite these fluctuations, the overall trend in the regression slope depicts a moderately significant decline over time among Asians (parameter estimate = -0.99, $p = 0.03$) (Table 2). Hispanics are the only population group with an increasing trend in TB incidence rate, equivalent to 3.2 cases of TB per $10^5$ person-years. From a low of 4.2 cases per $10^5$ person-years in 1982, the incidence rate in the Hispanic population reached a peak of 68.6 cases per $10^5$ person-years in 1999. The only other group showing an increasing trend is foreign-born persons, among whom TB incidence increased at the rate of 1.2 cases per $10^5$ person-years (Table 2). Compared with U.S.-born persons, foreign-born persons have a 36% higher risk for TB (Table 1). Those who have lived in the United States for <5 years constitute most of TB cases in foreign-born persons (62.2%). However, blacks still have about twice the risk of foreign-born persons (relative risk [RR] = 1.83; 95% confidence interval [95% CI] = 1.66-2.03).

Information about mode of administration of TB treatment was available in 91% of all cases. The proportion of TB patients receiving DOT (both exclusively and in combination with self-administered therapy) increased substantially, from 44.4% in 1993 to 94.7% by 1999 ($p < 0.0001$) (Figure 2). A high correlation was observed between declining rates of TB in the state and the increase in the proportion of TB patients receiving DOT ($r = -0.95$, $p = 0.0008$) (Figure 3). DOT was also found to correlate with completion of therapy over time ($r = 0.64$, $p = 0.1$).

Table 2. Trends in incidence rates of TB by selected variables, North Carolina, 1982–1999

| Variable | Parameter estimate[a] | p-value |
|---|---|---|
| Sex | | |
| Female | -0.24 | 0.0001 |
| Male | -0.55 | 0.0001 |
| Age group | | |
| <15 | -0.033 | 0.19 |
| 15-24 | -0.027 | 0.59 |
| 25-44 | -0.113 | 0.014 |
| 45-64 | -0.60 | 0.0001 |
| ≥65 | -1.41 | 0.0001 |
| Race | | |
| White | -0.21 | 0.0001 |
| Black | -1.11 | 0.0001 |
| Asian | -0.99 | 0.03 |
| Native American | -0.024 | 0.19 |
| Ethnicity | | |
| Non-Hispanic | -0.47 | 0.0001 |
| Hispanic | 3.2 | 0.0001 |
| Country of origin[a] | | |
| Foreign-born | 1.2 | 0.0084 |
| US-born | -0.53 | 0.0011 |
| US-born (blacks only) | -1.61 | 0.0033 |

[a]This denotes the slope that reflects an increasing (if positive) or decreasing (if negative) trend.
[a]Data from 1993 to 1999.

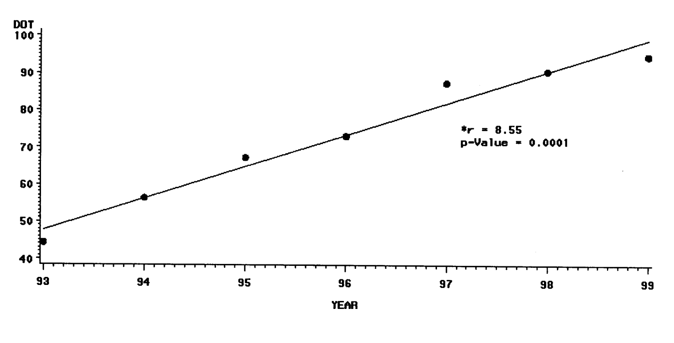

Figure 2. Proportion of tuberculosis patients receiving directly observed therapy (DOT) over time

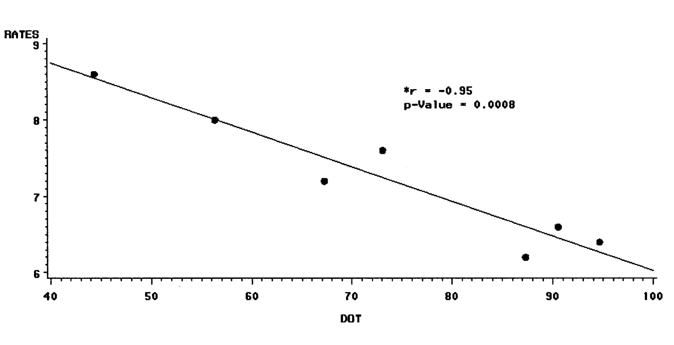

Figure 3. Correlation between incidence rates of tuberculosis (TB) and proportion of TB patients on directly observed therapy (DOT) over time

### Conclusions

Two decades ago North Carolina had among the worst TB records nationwide, ranking third in number of cases in 1980. Since that time, however, the incidence rate of the disease has decreased threefold, from 18.7 in 1980 to 6.2 per 100,000 in 1999. Two factors that may account for this significant decline are improved supervised therapy of patients and the related increase in the proportion of TB patients completing therapy. The proportion of TB patients receiving DOT in North Carolina has increased significantly over the study period and correlates strongly with the concomitant decline in the rate of TB over time ($r = -0.95$, $p = 0.0008$). In addition, completion rates, defined as the proportion of TB patients completing a prescribed anti-TB regimen, have also improved, correlating, although nonsignificantly, with increased reliance on DOT.

Our study is consistent with other reports that have demonstrated this direct link between improvement in DOT and decline in incident cases of TB (9-11). Other authors have also found DOT to be superior to unsupervised therapy, in addition to its role in curbing TB relapses (12). In addition, in persons predicted to be noncompliant because of psychiatric disease, alcoholism, drug addiction, substance abuse, and homelessness (13,14), DOT might be more successful than unsupervised therapy (15). Despite this evidence in support of DOT, it would be erroneous to conclude that DOT alone could have accounted for the observed significant reduction in TB cases in North Carolina. Factors such as adequate training and commitment of care providers and those involved in TB control programs, as well as overall administrative efficiency are important factors. Inadequacies in such a system or its functional collapse could render TB control and prevention ineffective regardless of improvement in DOT as was observed elsewhere (16). Another explanation could be the steady improvement in the living conditions of the population in general, leading to better housing, less overcrowding, and improved nutrition, and these factors are known to be inversely correlated with levels of TB disease.

We also found that men are two to three times more vulnerable to TB disease, in accordance with other reports (11,17,18). Given that the sex difference exists only between the ages of 25 to 64, in other words, the working age group, differential exposure to TB-associated risk factors, such as HIV infection, injecting drug use, alcohol abuse, and probably occupation, may be responsible for the consistent increased male risk for TB disease. Our data confirm that alcohol (odds ratio [OR] = 2.9, 95% CI = 2.27-3.719) and HIV infection (OR = 1.76, 95% CI = 1.295-2.392) account independently and significantly for the preponderance of TB disease among men. Therefore, in the ongoing battle to eliminate TB the presence of either condition, especially in men, should be considered as a potential marker for the possible concomitant presence of TB disease.

Age is an established risk factor for TB disease acquisition. Nationwide, the elderly ($\geq$65 years old) are the age group at the greatest risk, with incidence rates of 15 to 18 cases per 100,000, corresponding to a risk 4 to 9 times that of children (<15 years), the age group with the lowest risk (4-8,11,19). In North Carolina, TB risk among the elderly is 18 times that of children <15 years old. This age cohort represents people who must have been exposed to TB bacilli at a much younger age in the era of high TB disease rates in the United States. The disease remained latent as long as they enjoyed the enhanced immune status associated with younger age. Deterioration of the immune system with aging allows reactivation of dormant TB bacilli, leading to symptomatic disease.

The groups most affected by TB disease in North Carolina are racial and ethnic minorities: blacks, Hispanics, and Asians. Among Asians, the contribution of externally acquired TB infection may be the main source of TB disease. From 1993 to 1999, our data show that of 193 incident cases of TB reported in Asians, 181 cases (94%) are among foreigners. The main countries of origin are Vietnam (24%), the Philippines (15%), India (13%), Korea (9%), and Laos (7%). The same pattern is also observed among Hispanics, with 82% of cases in foreign-born persons, mostly from Mexico (78%). The high incidence of TB in these two minority communities is therefore externally acquired. A further source of concern is the sharp increase in incident cases of TB in the Hispanic population in the past 18 years, from 4.2 in 1982 to 68.6 per $10^5$ person-years in 1999, a phenomenon caused by the persistent increase in rates of TB in the Hispanic population, averaging 3.2 cases per $10^5$ person-years. In the state's black population, in contrast, only 85 TB cases (3.77%) of 2,252 recorded from 1993 to 1999 are accounted for by TB in foreign-born persons.

Nationwide, the proportion of U.S. TB cases attributable to foreign persons rose from 22% of the national total in 1986 to 37% a decade later (6). However, such a comparison based on shift in proportions may not be an appropriate scale for measuring the magnitude of TB disease in foreign-born persons, since a downward trend at one end of the balance (U.S.-born) implies an increase at the other end (foreign-born). Nevertheless, other studies have also found a two- to fourfold elevated risk for TB disease among immigrant residents compared with U.S.-born persons, tallying with similar reports of high TB incidence rates among immigrants (20-23). Although in North Carolina foreign-born persons have a 36% higher risk for TB disease than the U.S.-born, this elevation in risk is moderate compared with the national average risk for foreign-born persons (24). Indeed, our data show that U.S.-born blacks have an 83% higher risk for TB disease than foreign residents in the study area (RR 1.83; 95% CI 1.66-2.03).

The overall incidence of TB disease has significantly decreased over the past decade in North Carolina. The level of TB disease, however, is still very high among ethnic minorities (blacks, Hispanics, and Asians). Although rates of TB are declining in all other population subgroups, rates among foreign-born and Hispanic persons are increasing. The elimination of TB in the state may only be achieved by addressing the elevated rates in these groups, as well as the increase in incident cases among foreigners and Hispanics.

Dr. Salihu is a physician with special interest in population-based epidemiologic research. He also holds a PhD in epidemiology and biostatistics.

### References

1. Raviglione MC, Snider DE, Kochi A. Global epidemiology of tuberculosis. JAMA 1995;273:220-6.
2. Centers for Disease Control and Prevention. Tuberculosis morbidity: United States, 1995. MMWR Morb Mortal Wkly Rep 1996;45:365-70.

3. Centers for Disease Control and Prevention. Case definition for public health surveillance. MMWR Morb Mortal Wkly Rep 1996;45:171.
4. Centers for Disease Control and Prevention. Expanded surveillance and tuberculosis morbidity: United States, 1993. MMWR Morb Mortal Wkly Rep 1994;43:361-6.
5. Centers for Disease Control and Prevention. Tuberculosis morbidity: United States, 1994. MMWR Morb Mortal Wkly Rep 1995;44:387-95.
6. Centers for Disease Control and Prevention. Tuberculosis morbidity: United States, 1996. MMWR Morb Mortal Wkly Rep 1997;46:695-700.
7. Centers for Disease Control and Prevention. Tuberculosis morbidity: United States, 1997. MMWR Morb Mortal Wkly Rep 1998;47:253-7.
8. Centers for Disease Control and Prevention. Progress toward the elimination of tuberculosis—United States, 1998. MMWR Morb Mortal Wkly Rep 1999;27:732-6.
9. Chaulk CP, Moore-Rice K, Rizzo R, Chaisson ER. Eleven years of community-based directly observed therapy for tuberculosis. JAMA 1995;274:945-51.
10. Frieden TR, Fujiwara PI, Washko RM, Hamburg MA. Tuberculosis in New York City: turning the tide. N Engl J Med 1995;333:229-33.
11. Liu Z, Shilkret KL, Tranotti J, Freund CG, Finelli L. Distinct trends in tuberculosis morbidity among foreign-born and US-born persons in New Jersey, 1986 through 1995. Am J Public Health 1998;88:1064-7.
12. Weis SE, Slocum PC, Blais FX, King B, Nunn M, Matney B, et al. The effect of directly observed therapy on the rates of drug resistance and relapse in tuberculosis. N Engl J Med 1994;330:1179-84.
13. Blackwell B. Drug therapy: patient compliance. N Engl J Med 1973;289:249-52.
14. Sumartojo E. When tuberculosis treatment fails: a social account of patient adherence. Am Rev Respir Dis 1993;147:131-20.
15. McDonald RJ, Memon AM, Reichman LB. Successful supervised ambulatory management of tuberculosis treatment failures. Ann Intern Med 1982;96:297-302.
16. Goldstein A. D.C. shows jump in TB cases: study blasts city's efforts to track, fight disease. The Washington Post 1996 August 2; Metro C1.
17. Sotir MJ, Parrott P, Metchock B, Bock NN, McGowan JE, Ray SM, et al. Tuberculosis in the inner city: impact of a continuing epidemic in the 1990s. Clin Infect Dis 1999;29:1138-44.
18. Barnes P, Yang Z, Preston-Martin S, Pogoda JM, Jones BE, Otaya M, et al. Patterns of tuberculosis transmission in Central Los Angeles. JAMA 1997;278:1159-63.
19. McKenna MT, McCray E, Onorato IM, Castro KG. The epidemiology of tuberculosis among foreign-born persons in the United States, 1986 to 1993. N Engl J Med 1995;332:1071-6.
20. Enarson DA, Wang JS, Dirks JM. The incidence of active tuberculosis in a large urban area. Am J Epidemiol 1989;129:1268-76.
21. Raviglione MC, Sudre P, Rieder HL, Spinaci S, Kochi A. Secular trends of tuberculosis in western Europe. Bull World Health Organ 1993;71:297-306.
22. Stehr-Green JK. Tuberculosis in New Zealand, 1985-90. N Z Med J 1992;105:301-3.
23. Medical Research Council Cardiothoracic Epidemiology Group. National survey of notifications of tuberculosis in England and Wales in 1988. Thorax 1992;47:770-5.
24. Zuber PLF, McKenna MT, Binkin NJ, Onorato IM, Castro KG. Long-term risk of tuberculosis among foreign-born persons in the United States. JAMA 1997;278:304-7.
